# *Carex muskingumensis* and Osmotic Stress: Identification of Reference Genes for Transcriptional Profiling by RT-qPCR

**DOI:** 10.3390/genes11091022

**Published:** 2020-08-31

**Authors:** Magdalena Sozoniuk, Marzena Parzymies, Danuta Kozak, Bairam Solomon Ismael

**Affiliations:** 1 Institute of Plant Genetics, Breeding and Biotechnology, University of Life Sciences in Lublin, 20-612 Lublin, Poland; magdalena.sozoniuk@up.lublin.pl (M.S.); 2 Institute of Horticultural Production, Subdepartment of Ornamental Plants and Dendrology, University of Life Sciences in Lublin, 20-612 Lublin, Poland; dkozak@autograf.pl (D.K.); bairam_mr@yahoo.com (B.S.I.)

**Keywords:** RT-qPCR, reference genes, sedges, salinity, drought

## Abstract

*Carex muskingumensis* is a highly valued perennial ornamental grass cultivated worldwide. However, there is limited genetic data regarding this species. Selection of proper reference genes (RGs) for reverse transcription quantitative PCR (RT-qPCR) data normalization has become an essential step in gene expression analysis. In this study, we aimed to examine expression stability of nine candidate RGs in *C. muskingumensis* plants, subjected to osmotic stress, generated either by salinity or PEG treatment. The identification of genes exhibiting high expression stability was performed by four algorithms (geNorm, NormFinder, BestKeeper and deltaCt method). The results showed that the combination of two genes would be sufficient for reliable expression data normalization. ADP (ADP-ribosylation factor) and TBP (TATA-box-binding protein) were identified as the most stably expressed under salinity treatment, while eIF4A (eukaryotic initiation factor 4A) and *TBP* were found to show the highest stability under PEG-induced drought. A set of three genes (*ADP*, *eIF4A* and *TBP*) displayed the highest expression stability across all experimental samples tested in this study. To our best knowledge, this is the first report regarding RGs selection in *C. muskingumensis*. It will provide valuable starting point information for conducting further analyses in this and related species concerning their responses to water shortage and salinity stress.

## 1. Introduction

*Carex* L. is the largest and the most widespread genus in the *Cyperaceae* family [1]. *Carex muskingumensis* Schwein., also known as Muskingum sedge or Palm sedge, is a highly valued perennial ornamental grass cultivated worldwide. It is used as a ground cover or planted in flowerbeds or containers. The species is rather easy to grow in an average, medium to wet soil, in full sun or part shade [2]. 

During its life cycle plants are exposed to various environmental stresses. Water deficits, as well as soil salinization are currently becoming increasingly a problem in various regions of the world. Both drought and high salinity disrupt osmotic balance in plant cells and in consequence limit plant growth and development [3,4]. Therefore, knowledge of plants molecular response to such conditions is of great importance. In order to better understand plants tolerance adaptation mechanisms, many studies have focused on monitoring changes in genes expression under PEG-induced drought [5,6,7] or salinity treatments [8,9,10]. 

Reverse transcription quantitative PCR (RT-qPCR) constitutes commonly used approach in gene expression studies. However, in order to avoid erroneous results and consequently incorrect conclusions this technique requires proper data normalization. The necessity for reliable RT-qPCR data normalization has been emphasized in MIQE guidelines by Bustin et al. 2009 [11]. An accurate data analysis can be achieved with the help of reference genes (RGs)” [12]. An ideal RG would be a gene that displays constitutive, stable expression across different species regardless of tissue types, developmental stages and experimental conditions [13]. The ideal RG, however, does not exist or at least has not yet been identified. Previous studies have shown that a RG, which is considered to be stable under given experimental conditions often exhibits variable expression under different treatments [14,15,16]. The same have been demonstrated for different organs of the same species [17,18,19] or different species subjected to the same treatment [20,21]. Consequently, it is necessary to perform RG selection within each gene expression study. This step is usually conducted on the panel of candidate genes, that have previously been reported as stable under similar conditions and/or in related species. Commonly used RGs include genes encoding actin (*ACT*), tubulin (*TUB*), glyceraldehyde-3-phosphate dehydrogenase (*GAPDH*), elongation factor 1-α (*EF1a*) or ubiquitin (*UBQ*) [22]. Since the identification of appropriate RGs for RT-qPCR analysis has become fundamental in gene expression studies, several software programs, which use distinct algorithms facilitating this step have been developed, such as geNorm [23], NormFinder [24], BestKeeper [25] as well as deltaCt method [26].

The information on sedges’ molecular responses to osmotic stress and their adaptation mechanisms is limited [27,28,29]. To address this issue, our study aims at identifying best RGs for *C. muskingumensis* under salt and drought stress. Here, we determine expression stability of nine candidate RGs encoding actin 7 (ACT7), ADP-ribosylation factor (ADP), elongation factor 1-α (EF1a), eukaryotic initiation factor 4A (eIF4A), glyceraldehyde-3-phosphate dehydrogenase (GAPDH), phosphoenolpyruvate carboxylase-related kinase 1 (PEPKR1), SAND family protein (SAND), tubulin α (TUBa) and TATA-box-binding protein (TBP) with the use of four different algorithms. The current study will provide useful information for conducting further analysis in this and related species concerning their responses to water shortage and salinity stress. 

## 2. Materials and Methods

### 2.1. Experiment Design

The plant material were *C. muskingumensis* tufts obtained from a stable tissue culture grown on the solidified Murashige and Skoog (MS) [30] medium supplemented with kinetin (KIN) in concentration of 2.5 mg·dm^−3^ and indole-3-acetic acid (IAA) in concentration of 0.25 mg·dm^−3^. The pH was adjusted to 5.7. Explants for the experiment were 3–4 cm long single shoots with 4–5 leaves. The explants were placed on the MS medium supplemented with 1.5% or 3% of NaCl for salinity treatment and 2% or 8% of PEG (polyethylene glycol) for drought treatment. The medium without NaCl or PEG (0%) was treated as a control. Each treatments consisted of 4 flasks with 5 shoots. The explants were grown in the temperature of 22 °C day/20 °C night and 16 h photoperiod with 35 µmol·m^−2^·s^−1^ light intensity.

### 2.2. The RNA Extraction and Reverse Transcription

The RNA extraction was conducted after 7 days of cultivation, in four biological replicates for each treatment. Immediately after harvesting leaves from the treated and control plants, the tissue (100 mg) was homogenized in liquid nitrogen using mortar and pestle. Total RNA isolation was conducted with the use of TRIzol reagent (invitrogen, Carlsbad, CA, USA) in accordance with the manufacturer’s instructions. In order to prevent RNA degradation ribonuclease inhibitor (EURx, Gdansk, Poland) was added to all samples to final concentration of 1 U/µL. The RNA concentration was assessed with NanoDrop 2000 spectrophotometer (Thermo Scientific, Wilmington, DE, USA). The integrity of RNA samples was analysed by the means of electrophoresis in 2% agarose gel stained with ethidium bromide. 

Prior to cDNA synthesis, genomic DNA was removed by DNase I (EURx) treatment. The reverse transcription was performed with NG dART RT kit (EURx) according to the supplier’s recommendations. The components of reaction mixture were as following: 0.5 µg RNA, 4 µL of 5 × NG cDNA Buffer, 1 µL of 50 µM Oligo(dT)_20_ primer, 1 µL of NG dART RT mix and RNase-free water to final volume of 20 µL.

### 2.3. RT-qPCR Reactions

Nine genes (*ACT7*, *ADP*, *EF1a*, *eIF4A*, *GAPDH*, *PEPKR1*, *SAND*, *TUBa*, *TBP*) that are more or less frequently used as internal controls for RT-qPCR data normalization were chosen as candidates for RGs selection. Since no sequences for *C. muskingumensis* were available, *Carex rigescens* sequences deposited in GeneBank or SRA database (Sequence Read Archive accession No. SRX2755644) [27] were used for primer designing. *Arabidopsis thaliana* and *Zea mays* nucleotide sequences were used as query sequences for performed BLASTN search. Primers for RT-qPCR were designed with PrimerBLAST tool [31] (Table S1). 

The RT-qPCR reactions were based on SYBR Green chemistry. The reaction mixture (final volume of 25 µL) contained 10 ng of cDNA, 1 × SG/ROX qPCR Master Mix (EURx), 400 nM of each primer and 0.25 U uracil-*N*-glycosylase. The reactions were performed according to the following cycling program: 2 min at 50 °C, 10 min at 95 °C, 40 cycles of 15 s at 94 °C, 30 s at 60 °C and 30 s at 72 °C. All RT-qPCR reactions were conducted on a QuantStudio 3 apparatus (Applied Biosystems, Carlsbad, CA, USA). In order to verify the specificity of PCR products melting curve analysis was performed after each run with continuous data collection from 60 °C to 95 °C. The reactions were performed in four biological replicates with two technical replicates for each sample. No-template controls were included in analyses. Standard curves were generated from serial dilution of pooled cDNA. The PCR efficiencies for each primer pair were determined according to the equation [10^(1/−S)^ − 1] × 100%, where S represents the standard curve slope value [25]. 

### 2.4. Analysis of Gene Expression Stability

The expression stability of tested genes was estimated using following algorithms: geNorm [23], NormFinder [24], BestKeeper [25] and deltaCt method [26]. According to the requirements, performed analyses were based, either on untransformed Cq values (for BestKeeper and deltaCt method) or relative quantities (for geNorm and NormFinder). 

For the validation of selected reference genes, the expression level of target gene ascorbate peroxidase (*APX*) was analyzed under tested experimental conditions. The expression data of *APX* was normalized using most stable and least stable RGs according to the obtained results. The RT-qPCR amplification conditions were the same as those described above. The relative expression level of the target gene was calculated using the 2^−∆∆Ct^ method.

## 3. Results

### 3.1. Growth of Shoots

**Figure 1:**
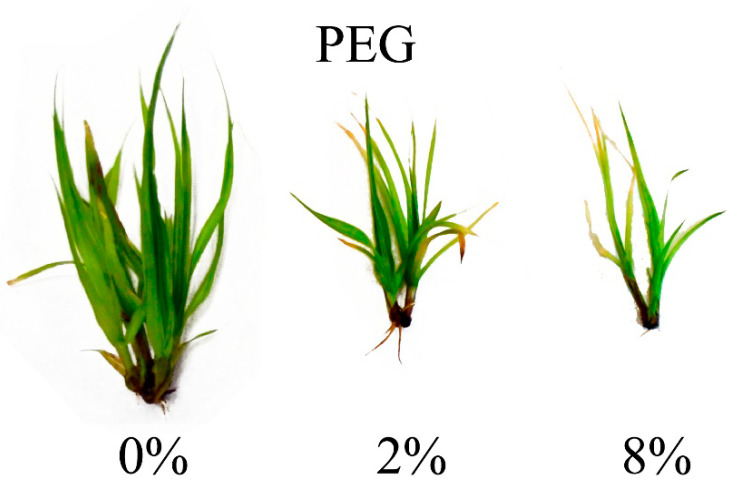
Growth of explants on the medium supplemented with PEG after 4 weeks of cultivation.

**Figure 2:**
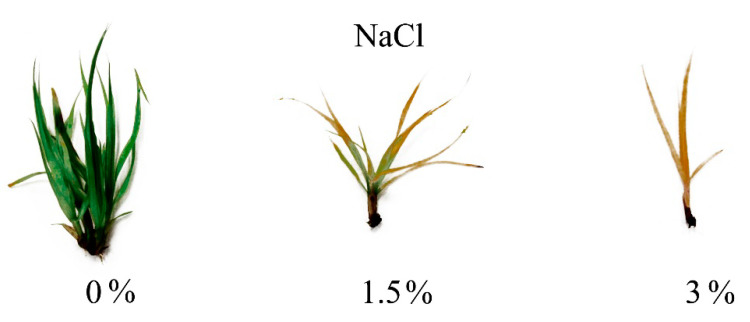
Growth of explants on the medium supplemented with NaCl after 4 weeks of cultivation.

*C. muskingumensis* shoots cultivated on the medium supplemented with PEG or NaCl had visible symptoms of the negative influence of the substances used. Explants grown on media, supplemented with PEG, were smaller than the control ones. It was noted that the higher concentration of PEG the smaller plants were obtained (Figure 1). Explants treated with NaCl were definitely damaged after the first week of treatment. It was observed that leaves started to turn pale green after the first week of cultivation, changing into brown after the second and the third ones. On the medium supplemented with 8% NaCl most explants died after four weeks of cultivation (Figure 2).

### 3.2. Candidate Reference Genes—Efficiency and Specificity of Amplification 

Nine genes (*ACT7*, *ADP*, *EF1a*, *eIF4A*, *GAPDH*, *PEPKR1*, *SAND*, *TUBa*, *TBP*) were chosen as candidate RGs for normalization of gene expression data in *C. muskingumensis* plants subjected to NaCl and PEG treatments. Confirmation of designed primer pairs suitability for RT-qPCR analysis was based on the analysis of standard curves performed on pooled cDNA and melting curves. Determined reaction efficiency, slope and regression coefficient (*R*^2^) are shown in Table S2. Amplification efficiency of each primer pair was above 90% and it ranged from 95.6% (*ADP*) to 111.2% (*PEPKR1*). Regression coefficients were all above 0.99 except for the *EF1a* where it reached 0.986. Specificity of each designed primer pair was confirmed by a single peak on dissociation curves (Figure S1).

#### 3.2.1. Reference Gene Selection 

Nine candidate RGs were screened among all experimental samples in order to find the most stable ones in tested material. Tested genes displayed variation in their expression across all of the samples with Cq value ranging from 21.83 to 32.82 (Table S3). Out of all candidate RGs *ACT7* had the highest expression with mean Cq value of 23.11, while *PEPKR1* showed the lowest expression level with mean Cq value of 30.77. The widest range of expression (Cq difference of almost 5 cycles) suggesting low stability level was observed for *TUBa* gene. 

The expression stability of selected candidate RGs was investigated with four different algorithms—geNorm, NormFinder, BestKeeper and deltaCt method. The analyses were conducted for three different datasets, that was data obtained from NaCl experiment, data obtained from PEG experiment and combined data of two abovementioned experiments.

#### 3.2.2. geNorm Analysis

**Figure 3:**
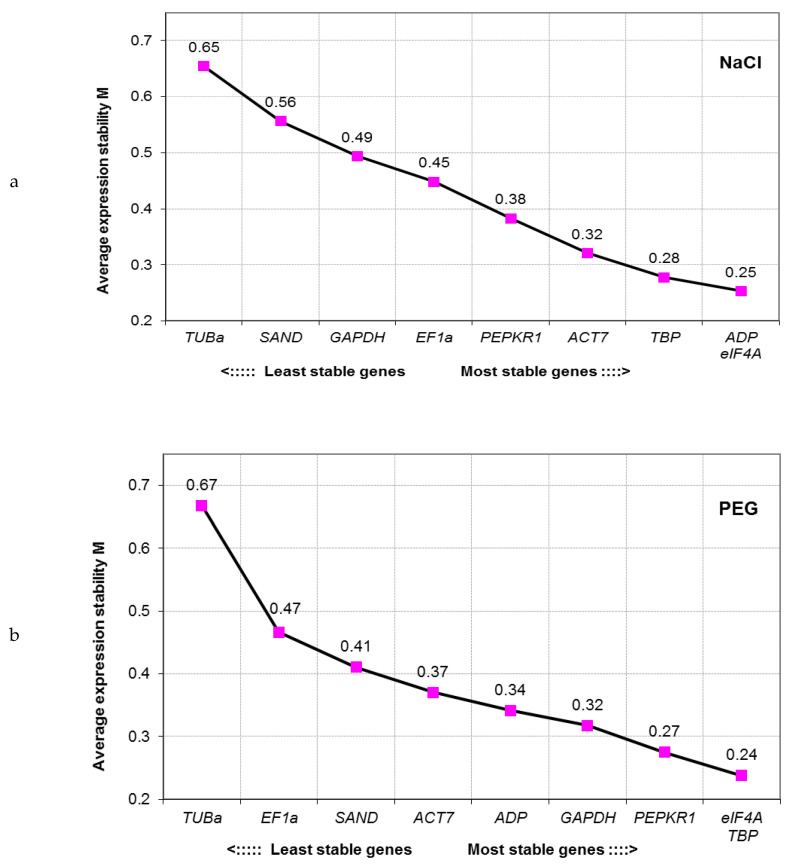

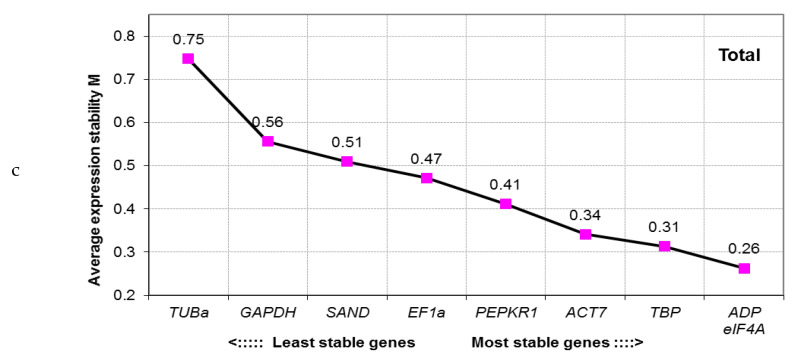
Average expression stability M of all remaining control genes after stepwise exclusion of the least stable reference genes according to the geNorm algorithm. The lower the M value the more stable is the gene’s expression in tested samples. Analysis was performed separately for (**a**) NaCl treated samples (NaCl), (**b**) PEG treated samples (PEG) and (**c**) all samples combined together (Total).

The geNorm algorithm ranks the candidate RGs according to the average expression stability value (M value). The lower the M value the more stable is the gene’s expression. The M value threshold for RG selection was established at 1.5 by Vandesompele et al. [23]. All tested candidate genes in all analyzed datasets (Figure 3) had the M value lower than the 1.5 cutoff. The same three genes (*eIF4A*, *ADP* and *TBP*) displayed the most stable expression in combined dataset (PEG plus NaCl samples) as in NaCl experiment alone. The most stable RGs, found in PEG-treated samples, were *eIF4A*, *TBP* and *PEPKR1*. *TUBa* exhibited the least stable expression in all experimental conditions (regardless of the NaCl and PEG samples being analyzed separately or together).

#### 3.2.3. NormFinder Analysis

NormFinder algorithm generates a stability value (SV) for each candidate RG including in its estimations both intra-group and inter-group variation in the sample set. The lower the SV, the more stable is the gene’s expression [24]. The best-scoring genes for samples subjected to NaCl stress were *ADP*, *TBP* and *ACT7*. In PEG treated samples the highest stability was displayed by *PEPKR1*, *eIF4A* and *TBP* (Table 1). When all of the sample sets were analyzed together the NormFinder ranked *TBP*, *ADP* and *eIF4A* as the most stable RGs for data normalization. Noteworthy, *TBP* gene was among three most stable RGs regardless of what data set was used for the calculations. On the other hand, the *TUBa* gene displayed the lowest expression stability in all of the data sets analyzed. 

#### 3.2.4. BestKeeper Analysis

**Figure 4:**
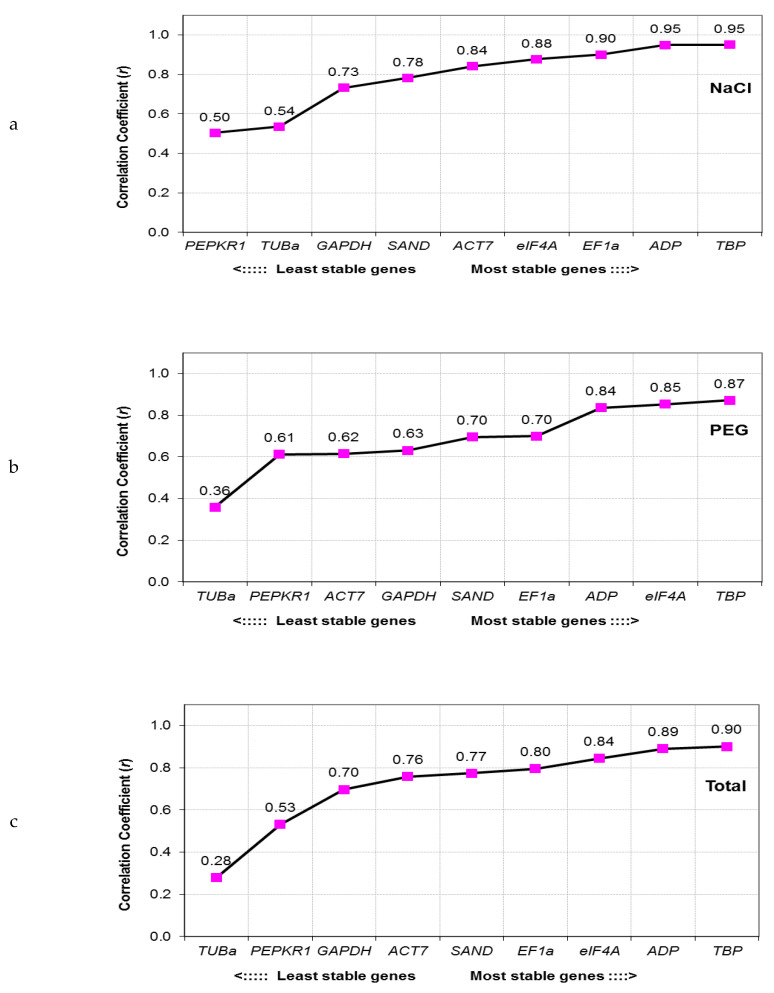
Determination of the most stable reference genes based on their correlation coefficients (*r*) according to the BestKeeper algorithm. The higher the correlation coefficient, the more stable is the gene’s expression. Analysis was performed separately for (**a**) NaCl treated samples (NaCl), (**b**) PEG treated samples (PEG) and (**c**) all samples combined together (Total).

BestKeeper software calculates its statistics based on Cq values. The best RGs are identified by assessing correlation coefficient (*r*) of each individual gene with the BestKeeper index (the geometric mean of all candidate genes). The most stably expressed genes are those exhibiting the highest coefficient of correlation [25]. Out of all tested candidate RGs, *TBP* was found to be the most stable, regardless of the treatment (Figure 4). High expression stability was also observed for *ADP*, *eIF4A* and *EF1a*, however, with interchanging ranking positions. Highest variation, on the other hand, was consistently displayed by *PEPKR1* and *TUBa* genes.

#### 3.2.5. deltaCt Method

The deltaCt method is an approach that identifies the most stable RGs by comparing relative expression of pairs of genes within each sample. Low average standard deviation (mean SD) reflects the low level of variability [26]. Based on the results generated from deltaCt method, highest stability of expression in all datasets was displayed by *ADP*, which was followed by either *TBP* or *eIF4A* (Table 2). Both *TUBa* and *PEPKR1* could be considered as least stable in tested material, as they were characterized by the highest mean SD. 

#### 3.2.6. Determination of Reference Genes Optimal Number 

**Figure 5:**
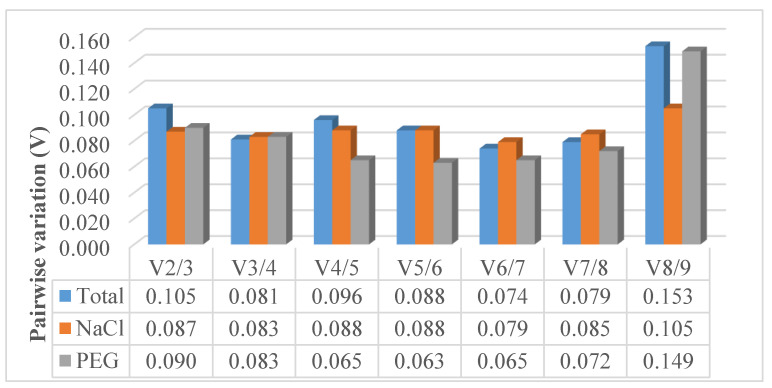
Determination of optimal number of reference genes calculated by geNorm. Pairwise variation (V_n_/V_n+1_) below 0.15 indicates no significant contribution made by inclusion of additional reference gene. Analysis was performed for NaCl treated samples (NaCl), PEG treated samples (PEG) and all samples combined together (Total).

The geNorm algorithm, additionally to ranking candidate RGs from the most to the least stable, allows for the determination of their optimal number for data normalization. The optimal number of RGs can be calculated by pairwise variation (V_n_/V_n+1_). If pairwise variation is below 0.15, the inclusion of additional RG does not make any significant contribution to the data analysis. In this study, V_2/3_ was lower than the threshold value of 0.15 (Figure 5) thus indicating that using just two best-performing RGs would be sufficient for expression data normalization. Nevertheless, Vandesompele et al. [23] and Pfaffl et al. [25] recommend the minimal use of at least three most stable RGs for reliable normalization. 

In the present study, when all samples were analyzed together, the same three genes were identified as the most stably expressed by all used algorithms, that is *ADP*, *eIF4A*, and *TBP*. However, their ranking positions slightly differed (Table 3). 

The *ADP* and *TBP* were among top three ranked genes in salinity treatment regardless of the calculation method. Both NormFinder and deltaCt method pointed out *ACT7* as third most stable gene in NaCl treatment, whereas geNorm and BestKeeper indicated *eIF4A*, or *EF1a*, respectively. 

According to all algorithms *eIF4A* and *TBP* were among the most stably expressed genes in drought treated plants. Moreover, *PEPKR1* was indicated as stably expressed during PEG-induced drought by both geNorm and NormFinder. However, according to the results generated by BestKeeper and deltaCt method, the *PEPKR1* gene showed high expression variability in this treatment. Instead, *ADP* was proposed as a gene showing relatively stable expression in this conditions. 

All algorithms indicated that both *SAND* and *GAPDH* showed medium to poor stability of expression. *ACT7* and *EF1a* mostly displayed intermediate level of stability. The *PEPKR1* gene, interchangeably with *TUBa*, were ranked by BestKeeper and deltaCt algorithms as two least stable genes in all sample sets. The geNorm and NormFinder invariably ranked *TUBa* at the last position, suggesting its high expression instability in tested conditions.

Additionally, the expression of target gene ascorbate peroxidase (*APX*) was analyzed, using most stable and least stable RGs, according to obtained results. Based on the comprehensive evaluation of RGs stability, we normalized *APX* expression in NaCl treated samples with *ADP*, *TBP*, combination of *ADP* + *TBP* and *TUBa*. PEG treated samples were normalized with *eIF4A*, *TBP*, combination of *eIF4A* + *TBP* and *TUBa*. The expression profile of *APX* under salinity stress was consistent when normalized with RGs showing high stability (Figure S2). However, a strong increase in *APX* expression could be observed when *TUBa* was used for data normalization. The expression of *APX* under drought treatment was at steady level with *eIF4A*, *TBP* or combination of *eIF4A* + *TBP* as a reference, whereas normalization with *TUBa* resulted in underestimation of *APX* expression (Figure S2). This confirms that selection of reliable RGs is crucial for proper target gene expression analysis. 

## 4. Discussion

The influence of specific treatments on candidate reference genes (RGs) expression has been well-presented in studies testing various stressing factors [16,17,18,32]. Depending on the conditions, applied candidate gene might exhibit, either stable or varied expression, thus, it might be useful or useless for data normalization. Since using unstable RGs may lead to erroneous results and incorrect conclusions, appropriate RT-qPCR data normalization has become necessity [13,22,33]. In this study, we investigated nine potential RGs (*ACT7*, *ADP*, *EF1a*, *eIF4A*, *GAPDH*, *PEPKR1*, *SAND*, *TUBa*, *TBP*) in terms of their stability in *C. muskingumensis* plants exposed to salinity and PEG-induced drought. 

The results of our experiment showed that the combination of *ADP* and *TBP* genes was the most suitable for normalization of data obtained from *C. muskingumensis* plants subjected to NaCl stress. Only one of the algorithms, used in this study, presented partially different results, selecting *ADP* with *eIF4A* as the most stable pair of genes. Still, *TBP* performed well and was identified as third in the ranking. More inconsistent results were found in *C. muskingumensis* plants exposed to PEG-induced drought. Here different algorithms selected different pairs of genes. Yet, each pair included *eIF4A*, thus, confirming its high stability under water deficit. Among other genes identified as highly stable were *TBP* (selected by two algorithms), *ADP* and *PEPKR1*. Partially inconsistent results, generated through various programs, are due to distinct statistical algorithms they use in stability calculations. Such differences in ranking positions were also reported in other studies [15,22,34].

More consistency in current study was observed in rankings obtained when both drought and salinity sample sets were considered together. Although with different ranking positions, the same three genes (*ADP*, *eIF4A*, *TBP*) were identified by all used algorithms as the most stable across tested conditions. Consequently, we believe that they constitute strong candidates for RT-qPCR data normalization. We suggest that these genes are included in the gene expression analyses of *C. muskingumensis* subjected to osmotic stress. 

The *ADP*, *eIF4A* and *TBP* have been previously identified as stable RGs in several plant species subjected to drought and salinity stresses. *ADP* performed well or very well in wild barley (*Hordeum brevisubulatum*) under different treatments that generated osmotic stress in cells [22]. *TBP* was found to be highly stable in drought treated celery (*Apium graveolens*) and moderately stable under salt stress [34]. The *eIF4A* was first in comprehensive rankings generated for perennial ryegrass (*Lolium perenne*) subjected to drought and high salinity stresses [35]. 

Study performed on close relative, *C. rigescens*, recommended *eIF4A* together with *PEPKR1* and *GADPH* to be preferentially selected for RT-qPCR analyses of that species [32]. To some extent, this is in concordance with our results, as *eIF4A* and *PEPKR1* ranked well or very well in the results generated by geNorm and NormFinder algorithms, particularly for PEG-treated samples. Both *eIF4A* and *PEPKR1* were among top three most stable genes in these rankings. Nevertheless, *GAPDH* did not perform as good as it might have been expected in the conditions of our experiment. 

Both *GAPDH* and *ACT* have been commonly used as RGs for RT-qPCR data normalization in plants [36,37,38,39,40]. However, it has been noticed that among many studies testing larger panels of potential RGs, few found *GAPDH* or *ACT* to show high stability of expression [13]. *ACT* and *GAPDH* exhibited variable expression in leaves of bermudagrass (*Cynodon dactylon*) under NaCl and PEG stress [18]. Likewise, both genes performed poorly in seashore paspalum (*Paspalum vaginatum*) exposed to drought and salinity [19]. Furthermore, *GAPDH* was reported to be least stable in annual ryegrass (*Lolium multiflorum*) under abovementioned stresses [16], while *ACT* was least stable in durum wheat (*Triticum durum*) subjected to drought [33].

In our study, *ACT7* performed slightly better than *GAPDH* in terms of expression stability in NaCl experiment, contrary to PEG-induced drought experiment, where the situation was reversed. Both genes, however, are considered to be far from optimal for *C. muskingumensis* data normalization, as they showed rather medium to poor expression stability in most rankings. Moreover, according to most of the algorithms *TUBa*, which is another ‘traditional’ RG, proved to be the least stable of all tested RGs in conditions of our study. High variability of *TUB* expression in response to drought and salt treatment was also reported in leaves of creeping bentgrass (*Agrostis stolonifera*) [17], as well as leaves of bermudagrass (*C. dactylon*) [18].

It has been agreed that RGs should be selected individually for each experiment [12,15,16]. However, prior to performing RGs selection, the type of candidate genes tested should be carefully considered. Apart from using a few ‘traditional’ RGs (such as *GAPDH*, *ACT*, *TUB*, *EF1a*), it is worth analyzing less frequently used genes or even novel genes, as they may turn out to be more stable under given conditions. For instance, Dudziak et al. [41] analyzed expression stability of five traditional RGs together with five novel candidate genes in common wheat (*Triticum aestivum*) lines subjected to drought stress. Both geNorm and NormFinder algorithms found novel gene, CJ705892, to be the most stable. Similarly, Liu et al. [16] tested six common RGs together with four novel candidate genes in annual ryegrass (*L. multiflorum*). They found that under drought stress one of the ‘traditional’ RGs (*eIF4A*) was the most stable. However, under saline-alkali stress highest stability was exhibited by one of the novel genes (Unigene14912). Therefore, while satisfactory results might be obtained by using conventional RGs (as it was done within this study), considering some alternatives might bring about valuable outcomes. 

The fact that gene expression analysis using RT-qPCR technique should be preceded by identification of most stable RGs within its sample set is nonnegotiable [12,13]. However, the results of other studies testing panels of potential RGs in the same, or closely related, species can provide valuable starting point information. Such studies might indicate which candidate RGs are worth being tested for expression stability. This is especially true for non-model organisms that are not yet well characterized [13]. 

To our best knowledge, apart from our investigation, RGs selection has been reported in the family *Cyperaceae* only in *C. rigescens* [32]. In the order *Poales*, such studies were conducted most commonly in the family *Poaceae*. In BOP clade evaluation of RGs was reported frequently in rice, wheat and barley and concerned various treatments (e.g., cold, heat, salinity, drought, submergence, heavy metals, hormone treatment, biotic stresses [14,22,33]). Other species from BOP clade, which were tested in respect to RGs stability belong to such genera as *Agropyron* [42], *Agrostis* [17], *Avena* [43], *Bambusa* [44], *Brachypodium* [45], *Elymus* [46], *Lolium* [16], *Poa* [47] and *Stipa* [48].

In PACMAD clade identification of stable RGs was reported several times in such genera as *Eleusine* [49], *Cynodon* [18] and *Setaria* [50].

Our experiment identified most stable RGs in *C. muskingumensis* plants subjected to osmotic stress. The data suggests that *ADP* and *TBP* would be the most suitable to use in salinity studies, while *eIF4A* in combination with *TBP* could be used in drought experiments. Across all tested conditions highest expression stability was displayed by *ADP*, *eIF4A* and *TBP*. To our best knowledge this is the first report regarding RGs selection in this species. The identification of stable RGs in *C. muskingumensis* will be helpful for accurate RT-qPCR data normalization and will facilitate further investigations of molecular mechanisms involved in its response to high salinity and drought. 

## Figures and Tables

**Table 1:** Stability analysis of candidate reference genes performed by NormFinder algorithm. The lower the stability value (SV) the more stable is the gene’s expression in tested samples. Analysis was performed for NaCl treated samples (NaCl), PEG treated samples (PEG) and all samples combined together (Total).

Rank	Total		NaCl		PEG	
	Gene	SV	Gene	SV	Gene	SV
1	*TBP*	0.187	*ADP*	0.138	*PEPKR1*	0.115
2	*ADP*	0.197	*TBP*	0.145	*eIF4A*	0.147
3	*eIF4A*	0.205	*ACT7*	0.170	*TBP*	0.220
4	*PEPKR1*	0.235	*eIF4A*	0.238	*ADP*	0.234
5	*ACT7*	0.278	*PEPKR1*	0.242	*GAPDH*	0.272
6	*EF1a*	0.305	*EF1a*	0.326	*SAND*	0.299
7	*SAND*	0.355	*SAND*	0.381	*EF1a*	0.326
8	*GAPDH*	0.452	*GAPDH*	0.432	*ACT7*	0.363
9	*TUBa*	0.794	*TUBa*	0.496	*TUBa*	0.793

**Table 2:** Candidate reference gene ranking according to deltaCt method. The lower the mean standard deviation (mean SD) value the more stable is the gene’s expression in tested samples. Analysis was performed for NaCl treated samples (NaCl), PEG treated samples (PEG) and all samples combined together (Total).

Rank	Total		NaCl		PEG	
	Gene	Mean SD	Gene	Mean SD	Gene	Mean SD
1	*ADP*	0.707	*ADP*	0.624	*ADP*	0.742
2	*TBP*	0.733	*TBP*	0.646	*eIF4A*	0.769
3	*eIF4A*	0.787	*ACT7*	0.765	*TBP*	0.787
4	*SAND*	0.834	*eIF4A*	0.777	*SAND*	0.824
5	*ACT7*	0.838	*SAND*	0.802	*ACT7*	0.923
6	*EF1a*	0.913	*GAPDH*	0.851	*EF1a*	0.971
7	*GAPDH*	0.999	*EF1a*	0.859	*GAPDH*	1.089
8	*PEPKR1*	1.076	*TUBa*	1.001	*PEPKR1*	1.188
9	*TUBa*	1.428	*PEPKR1*	1.033	*TUBa*	1.425

**Table 3:** Ranking of candidate reference genes stability according to all tested algorithms. Analyses were performed for NaCl treated samples (NaCl), PEG treated samples (PEG) and all samples combined together (Total).

Method	Stability (High→Low)								
	1	2	3	4	5	6	7	8	9
Total									
geNorm	*ADP/eIF4A*	*-*	*TBP*	*ACT7*	*PEPKR1*	*EF1a*	*SAND*	*GAPDH*	*TUBa*
NormFinder	*TBP*	*ADP*	*eIF4A*	*PEPKR1*	*ACT7*	*EF1a*	*SAND*	*GAPDH*	*TUBa*
BestKeeper	*TBP*	*ADP*	*eIF4A*	*EF1a*	*SAND*	*ACT7*	*GAPDH*	*PEPKR1*	*TUBa*
deltaCt	*ADP*	*TBP*	*eIF4A*	*SAND*	*ACT7*	*EF1a*	*GAPDH*	*PEPKR1*	*TUBa*
NaCl									
geNorm	*ADP/eIF4A*	*-*	*TBP*	*ACT7*	*PEPKR1*	*EF1a*	*GAPDH*	*SAND*	*TUBa*
NormFinder	*ADP*	*TBP*	*ACT7*	*eIF4A*	*PEPKR1*	*EF1a*	*SAND*	*GAPDH*	*TUBa*
BestKeeper	*TBP*	*ADP*	*EF1a*	*eIF4A*	*ACT7*	*SAND*	*GAPDH*	*TUBa*	*PEPKR1*
deltaCt	*ADP*	*TBP*	*ACT7*	*eIF4A*	*SAND*	*GAPDH*	*EF1a*	*TUBa*	*PEPKR1*
PEG									
geNorm	*eIF4A/TBP*	*-*	*PEPKR1*	*GAPDH*	*ADP*	*ACT7*	*SAND*	*EF1a*	*TUBa*
NormFinder	*PEPKR1*	*eIF4A*	*TBP*	*ADP*	*GAPDH*	*SAND*	*EF1a*	*ACT7*	*TUBa*
BestKeeper	*TBP*	*eIF4A*	*ADP*	*EF1a*	*SAND*	*GAPDH*	*ACT7*	*PEPKR1*	*TUBa*
deltaCt	*ADP*	*eIF4A*	*TBP*	*SAND*	*ACT7*	*EF1a*	*GAPDH*	*PEPKR1*	*TUBa*

## Supplementary Materials

The following are available online at https://www.mdpi.com/2073-4425/11/9/1022/s1. Figure S1: Melting curves for nine candidate reference genes, Table S1: Primer sequences for candidate reference genes, Table S2: Parameters derived from RT-qPCR analysis—slope, regression coefficient (R^2^), reaction efficiency and melting temperature of the amplicon (Tm), Table S3: Cq values of nine candidate reference genes under salinity and drought stresses.
