# Supplementary material for: Carex muskingumensis and Osmotic Stress: Identification of Reference Genes for Transcriptional Profiling by RT-qPCR
Source: Genes (Basel). 2020 Aug 31;11(9):1022. doi: 10.3390/genes11091022 (PMC7563777; doi:10.3390/genes11091022)
Supplement: Supplementary file 1 [file genes-11-01022-s001.docx]

**Supplementary Materials**


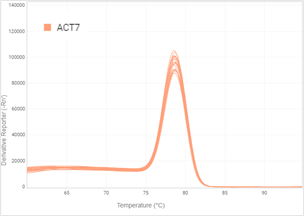

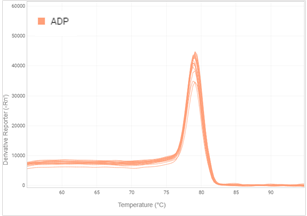

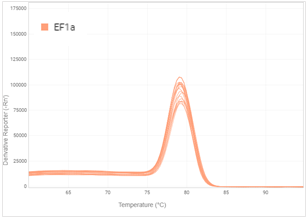

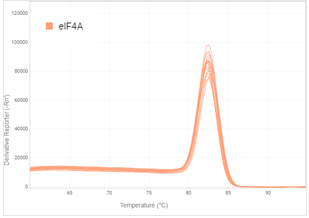

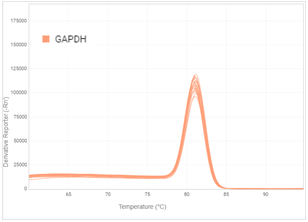

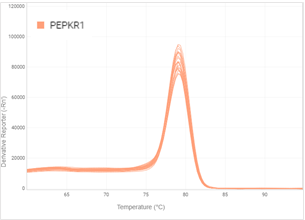

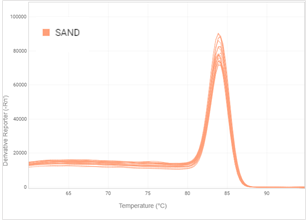

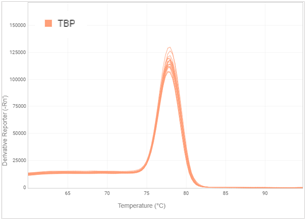

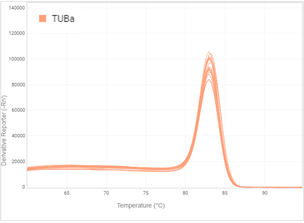


**Figure S1.** Melting curves for nine candidate reference genes.


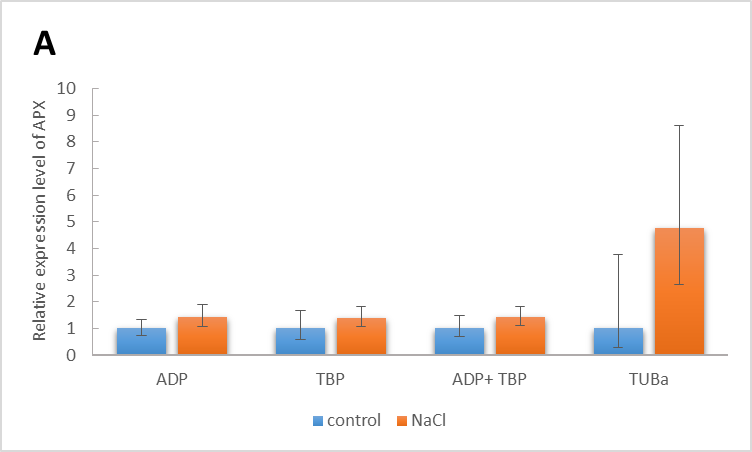

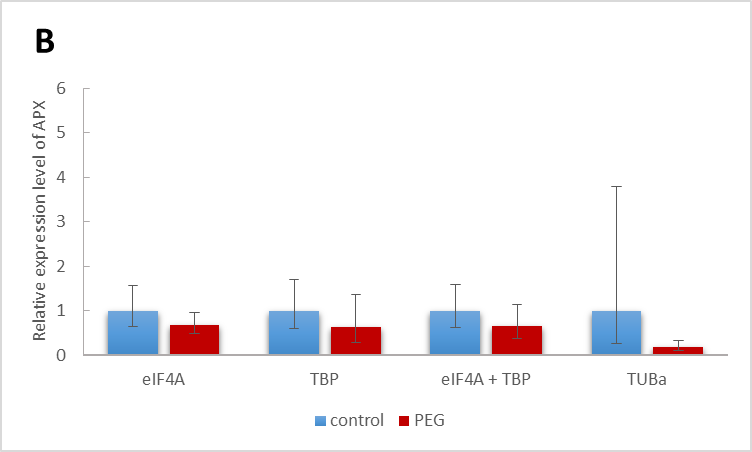


**Figure S2.** The relative expression level of *APX* under NaCl (A) or PEG (B) treatment normalized with selected RGs. The expression level for control was used as calibrator (relative expression level = 1). Bars represent standard deviation. Each treatment was analyzed in four biological replicates.

**Table S1.** Primer sequences for candidate reference genes.

| Gene symbol | Gene description | Accession number | Primer sequence 5’→3’ | Amplicon size (bp) |
| --- | --- | --- | --- | --- |
| *ACT7* | actin 7 | MF782856 | F: CATCCACGAGACAACCTAC | 85 |
|  |  |  | R: ACCACTGAGCACAATGTT |  |
| *ADP* | ADP-ribosylation factor | SRR5471218.22792464.2 | F: GGTATGGTGGTGACGATCTCT | 79 |
|  |  |  | R: TGATGGTGGGTCTCGATGC |  |
| *EF1a* | elongation factor 1-alpha | SRR5471218.21444324.2 | F: ACAAGCGTGTCATTGAACGT | 77 |
|  |  |  | R: AAGCACCCACGCGTACTTGA |  |
| *eIF4A* | eukaryotic initiation factor 4A | MF782862 | F: AGTGGAAGCTCGACACACTG | 123 |
|  |  |  | R: TGTGGTCACGACTACGCATC |  |
| *GAPDH* | glyceraldehyde-3-phosphate dehydrogenase | MF782858 | F: GTGCTTCCAGCTTTGAACGG | 150 |
|  |  |  | R: ATTGCCCTCAGACTCCTCCT |  |
| *PEPKR1* | phosphoenolpyruvate carboxylase-related kinase 1 | MF782861 | F: GTGGTGTCCCTCCTTTCTGG | 98 |
|  |  |  | R: AATAGAAGGCCATGGTGGGC |  |
| *SAND* | SAND family protein | MF782860 | F: CGAACCAAAACCGAACCGAC | 109 |
|  |  |  | R: GGTTCAACGGTGTTGTGCTC |  |
| *TUBa* | tubulin alpha | MF782857 | F: GCTTGGCAACTCTTGTTGGG | 132 |
|  |  |  | R: CCTGCACCAGTCTCGCTAAA |  |
| *TBP* | TATA-box-binding protein | MF782863 | F: GAAGGAAGCCAGCCTGTTGA | 124 |
|  |  |  | R: GGGCTTGCAAAGCAATAGCC |  |

**Table S2.** Parameters derived from RT-qPCR analysis - slope, regression coefficient (R^2^), reaction efficiency and melting temperature of the amplicon (Tm).

| Gene | Slope | R^2^ | Efficiency (%) | Tm (°C) |
| --- | --- | --- | --- | --- |
| *ACT7* | -3.234 | 0.997 | 103.791 | 78.5 |
| *ADP* | -3.433 | 0.995 | 95.568 | 79.5 |
| *EF1a* | -3.261 | 0.986 | 102.611 | 79 |
| *eIF4A* | -3.26 | 0.997 | 102.638 | 82.5 |
| *GAPDH* | -3.105 | 0.992 | 109.919 | 81 |
| *PEPKR1* | -3.079 | 0.992 | 111.237 | 79 |
| *SAND* | -3.359 | 0.994 | 98.492 | 84 |
| *TBP* | -3.274 | 0.996 | 102.031 | 78 |
| *TUBa* | -3.345 | 0.998 | 99.058 | 83 |

**Table S3.** Cq values of nine candidate reference genes under salinity and drought stresses.

| ACT7 | ADP | EF1a | eIF4A | GAPDH | PEPKR1 | SAND | TBP | TUBa |
| --- | --- | --- | --- | --- | --- | --- | --- | --- |
| 22.09 | 22.26 | 23.31 | 24.11 | 23.01 | 30.37 | 28.94 | 28.15 | 29.85 |
| 23.18 | 22.90 | 23.19 | 24.93 | 24.80 | 30.45 | 28.19 | 28.38 | 30.02 |
| 21.99 | 22.22 | 24.14 | 24.33 | 22.53 | 30.02 | 28.78 | 27.67 | 29.90 |
| 22.52 | 22.68 | 24.59 | 24.77 | 24.45 | 30.34 | 29.31 | 28.13 | 30.41 |
| 23.08 | 23.51 | 24.77 | 25.56 | 25.51 | 31.32 | 30.73 | 29.53 | 30.30 |
| 22.66 | 23.16 | 24.64 | 24.98 | 24.59 | 30.81 | 30.21 | 28.55 | 30.90 |
| 23.53 | 24.03 | 25.70 | 24.97 | 25.15 | 30.58 | 30.04 | 28.64 | 27.93 |
| 24.14 | 23.87 | 23.96 | 25.86 | 26.25 | 31.02 | 29.79 | 29.48 | 28.50 |
| 24.06 | 23.56 | 24.40 | 25.84 | 24.03 | 31.03 | 29.99 | 29.54 | 28.16 |
| 23.68 | 23.45 | 24.54 | 25.47 | 23.60 | 28.33 | 29.62 | 29.20 | 27.94 |
| 22.97 | 23.91 | 26.29 | 26.54 | 24.04 | 32.03 | 29.95 | 30.20 | 30.67 |
| 23.70 | 23.81 | 26.10 | 26.16 | 25.73 | 31.77 | 30.16 | 30.07 | 31.53 |
| 24.73 | 24.29 | 25.88 | 26.47 | 24.36 | 31.97 | 29.75 | 30.04 | 29.65 |
| 23.91 | 23.99 | 26.43 | 26.44 | 24.27 | 29.00 | 30.48 | 29.26 | 30.68 |
| 22.33 | 22.15 | 24.15 | 23.87 | 23.21 | 30.37 | 28.75 | 28.11 | 30.21 |
| 23.86 | 23.69 | 25.78 | 25.08 | 24.46 | 32.03 | 31.28 | 29.59 | 32.82 |
| 22.61 | 22.24 | 24.10 | 24.11 | 22.92 | 30.90 | 29.70 | 28.46 | 30.70 |
| 22.50 | 23.14 | 24.27 | 25.44 | 24.57 | 31.17 | 29.14 | 29.32 | 30.37 |
| 22.72 | 22.72 | 24.45 | 24.82 | 23.66 | 30.94 | 29.10 | 28.82 | 31.27 |
| 21.83 | 22.26 | 24.20 | 24.16 | 23.39 | 30.87 | 28.97 | 27.81 | 29.90 |
